# CD4+ Th17 Cells Discriminate Clinical Types and Constitute a Third Subset of Non Th1, Non Th2 T Cells in Human Leprosy

**DOI:** 10.1371/journal.pntd.0002338

**Published:** 2013-07-25

**Authors:** Chaman Saini, V. Ramesh, Indira Nath

**Affiliations:** 1 National Institute of Pathology (ICMR), Safdarjung Hospital Campus, New Delhi, India; 2 Department of Dermatology, Safdarjung Hospital Campus, New Delhi, India; University of California San Diego School of Medicine, United States of America

## Abstract

**Background:**

Patients with localized tuberculoid and generalized lepromatous leprosy show respectively Th1 and Th2 cytokine profile. Additionally, other patients in both types of leprosy also show a non discriminating Th0 cytokine profile with both interferon-γ and IL-4. The present study investigated the role of Th17 cells which appear to be a distinct subtype of Th subtypes in 19 tuberculoid and 18 lepromatous leprosy patients. Five healthy subjects with long term exposure to infection and 4 skin biopsies from healthy subjects undergoing cosmetic surgery were used as controls.

**Methodology/Principle Findings:**

An array of Th17 related primers for cytokines, chemokines and transcription factors was used in real time reverse transcribed PCR to evaluate gene expression, ELISA for cytokine secretion in the supernatants of antigen stimulated PBMC cultures and flow cytometry for establishing the phenotype of the IL-17, IL-21 producing cells.

**Conclusions/Significance:**

IL-17 isoforms showed significantly higher expression and release in supernatants of antigen stimulated PBMC cultures and dermal lesions of healthy contacts and tuberculoid leprosy as compared to lepromatous leprosy (p<0.003). This was further confirmed by Th17 associated transcription factor RORC, cytokines IL-21, IL-22, and IL-23, chemokines MMP13, CCL20, CCL22. Of interest was the association of IL-23R and not IL-6R with IL-17^+^ cells. The Th17 cells were CD4^+^ CCR6^+^ confirming their effector cell lineage. Polarized Th1 cytokines were seen in 3/7 tuberculoid and Th2 cytokines in 5/10 lepromatous leprosy patients. Of importance was the higher association of Th17 pathway factors with the non-polarized Th0 types as compared to the polarized Th1 and Th2 (p<0.01). Our study draws attention to a third type of effector Th cell that may play a role in leprosy.

## Author Summary

Leprosy caused by *Mycobacterium leprosy* continues to be a public health challenge in developing countries. It manifests as a leprosy spectrum with varied clinical forms of localized (tuberculoid) and generalized (lepromatous) disease. Consensus on the immunological basis for leprosy spectrum is lacking. T helper subsets of Th1 and Th2 based on mutually exclusive cytokines IFN-γ and IL-4 respectively were considered as a basis for the inverse relationship between T cell functions and antibody responses seen in this disease. However, there are some patients who show Th0 subset which is non discriminatory and produce both cytokines. In the present study, we provide evidence for the presence of the recently described third subset of Th17 in both antigen stimulated PBMC and skin lesions of tuberculoid leprosy. They appear to be associated with Th0 profile and may represent a third type of effector cell in leprosy.

## Introduction

Leprosy caused by *Mycobacterium leprae* continues to be a public health challenge in developing countries where total number of new cases reported in 2011 was 210,075 in spite of multi drug regimen and good public health practices [1]. Of this, South East Asia alone reported the highest number of new cases of 160,132 followed next by the Americas with 36,832. The causative agent is non-cultivable by conventional methods and infects mainly man and the nine banded armadillo. Clinical leprosy presents uniquely as a five point clinic-pathological spectrum with polar (TT) and borderline tuberculoid (BT) leprosy at one end with patients showing paucibacillary, hypoanesthetic, hypopigmented skin patches. At the other end of the spectrum lie polar (LL) and borderline lepromatous leprosy (BL) with generalized multibacillary skin lesions. In between lies borderline borderline (BB) leprosy which is unstable with patients moving towards tuberculoid or lepromatous presentations [2]. *M.leprae* is the only bacillus that infects peripheral nerves and shows earlier involvement in tuberculoid and relatively later pathology in lepromatous leprosy [3]. It is now well established that subjects with the localized disease have good T cell immunity to the pathogen and poor antibody responses whereas the converse is observed in lepromatous leprosy. The dichotomy between T cell and antibody responses appeared to be in consonance with the concept of Th1 and Th2 subsets of helper T cells with mutually exclusive cytokine signatures [4], [5]. Thus lepromatous leprosy was associated with IL-4, IL-5 and absence of IFN-γ in antigen stimulated PBMC cultures as well as skin lesions indicating Th2 polarization and tuberculoid leprosy in contrast was reported to show Th1 responses where IFN-γ, IL-2 were the predominant cytokines [6], [7], [8] . However, many patients in both types of leprosy also show concomitant presence of IL-4 and IFN-γ in antigen stimulated PBMC cultures indicating the presence of non polarized Th0 responses to the pathogen [6], [7], [9] and greater heterogeneity in cytokine producing cells at clonal level [10]. At present there is no clear consensus on the central mechanisms that underlie the leprosy spectrum or the antigen specific T cell anergy that is associated with lepromatous leprosy. The lack of a suitable animal model that mimics human leprosy limits our understanding of the events prior to the establishment of the clinical spectrum.

In addition to Th1 and Th2, a third subset of effector cells that produce IL-17 has been identified in the past decade and designated as Th17 [11], [12]. The primary role of this cell appears to be clearance of pathogen which has not been controlled by Th1 and Th2. Attention was first drawn to the pivotal role of this subset in experimental auto immune encephalitis and subsequently implicated in other infectious diseases [13] such as tuberculosis [14], [15], leishmaniasis [16], and fungi [17] as well as disorders such as cancers [18], [19], mucosal bowel disease [20] psoriasis [21] and vitiligo [22]. Some species related differences are evident in Th17 cells of mice and man [23]. The unique cytokine of Th17 is IL-17 which has several isoforms of which IL-17A and IL-17F appear to be important in regulating immune responses in autoimmunity [24]. Moreover other cytokines such as IL-21 [25], IL-22 [26] are also secreted by Th17 cells along with chemokine CCL20 [27]. In man, the transcription factor, retinoic acid related orphan nuclear hormone receptor C (RORC) is associated with Th17 cells and is a homologue of the murine RORγt [28]. Moreover, receptors IL-23R [29], IL-1R1 [30], chemokines CCR6 [31], [32] and CCR4 [32] are also expressed on Th17 cells . Some reports have demonstrated that human Th17 cells are able to produce both IL-17A and IFN-γ. IFN-γ production required IL-12 [33]. Th17 clones were seen to express IL12rβ2 in addition to IL-23R and Th1 related T-bet as well as RORC [34].

With a view to understanding the basis of the leprosy spectrum particularly in patients who showed non Th1 and non Th2 polarization we investigated the role of Th17 cells in tuberculoid and lepromatous leprosy patients at the two ends of the spectrum in both PBMC and chronic inflammatory/granulomatous skin lesions. We also compared the patients with the healthy clinically normal subjects who had been in constant contact with leprosy patients for 1–2 years (HC), using quantitative real time polymerase chain reaction (qPCR) for expression of genes in the Th17 pathway, ELISA for quantitation of cytokines and flowcytometry for identification of cell types in PBMC cultures stimulated with sonicated heat killed armadillo derived *M.leprae* antigen (ML). Of interest was the higher Th17 cell activity observed in HC and borderline tuberculoid subjects (BT) as compared to those with polar lepromatous leprosy (LL). Importantly, the association of Th17 cell signatures in leprosy patients was observed in patients who did not show the conventional Th1 and Th2 phenotypes but had a non polarized subset of CD4^+^T cells (Th0 type) which expressed and secreted IFN-γ and IL-4/IL-5 in antigen stimulated PBMC cultures.

## Methods

### Experimental strategy and rationale

In this study we considered the two clinical types of paucibacillary tuberculoid (BT) and multibacillary lepromatous leprosy (BL, LL) as reflecting natural relative resistance and susceptibility respectively to *M.leprae* infection. In addition, healthy subjects who had been in contact for 1–2 years with leprosy patients and had not developed the disease and considered be resistant to the disease were included as controls. In view of recent reports on Th17 in relation to resistance to intracellular pathogens [13], [17] and the lack of consensus on the mechanisms underlying anergy in leprosy, we investigated Th17 cell pathways using PBMC and skin lesions from patients and healthy skin from subjects undergoing cosmetic surgery. Expression of the genes in the pathway of Th17 as well as the identification of cell types expressing IL-17 and IL-21 was investigated using quantitative RT-PCR and flowcytometry respectively. ELISA was used to evaluate the secretion of relevant cytokines into the supernatants of *ex vivo* cultures of antigen stimulated PBMC.

### Ethics statement

The study protocol, informed consent forms in local language and all procedures were approved by the Institutional Ethical Committee of Safdarjung Hospital (No. 26-11-EC (25/31). Written informed consent was obtained from the patients after counseling and prior to taking blood samples and skin biopsies.

### Human subjects

37 newly diagnosed leprosy patients without history of anti-leprosy treatment (26 males, 11 females aged between 19–60 years) attending the Leprosy Clinics of the Department of Dermatology, Safdarjung Hospital, New Delhi were included in the study (Table 1). Leprosy type was determined by clinical and histological criteria on the basis of Ridley-Jopling classification [2]. Study group included 19 borderline tuberculoid (BT) and 18 polar lepromatous (LL) leprosy patients. Five healthy subjects who were house hold members in contact with leprosy patients for 1–2 years were included as controls (HC) since they showed no evidence of disease after continuing exposure to the same environment and in close contact with infected patients. In addition, normal skin samples (N) from 4 patients undergoing cosmetic surgery for burns were also included as controls.

**Table 1:** Clinical characteristics of freshly diagnosed, untreated leprosy patients and healthy contacts of patients and healthy skin (from cosmetic surgery) included in study.

Clinical type (number of subjects)	Age in years	Sex		Duration of disease (in months)	BI
		M	F		
BT (19)	19–59	13	6	1–36	0–1+
LL (18)	24–60	13	5	6–12	5–6+
HC (5)	22–40	3	2	-	-
Healthy skin (4)	22–28	4	-	-	-

BT: borderline tuberculoid leprosy, LL: polar lepromatous leprosy, as per Ridley Jopling classification [2], HC; healthy contacts exposed to leprosy patients to 1–2 years. BI: Bacillary Index (mean of six lesional sites). M; male, F; female. Healthy skin; patients undergoing cosmetic surgery for burns were also included as controls.

### Isolation of Peripheral Blood Mononuclear Cells (PBMC)

10 ml of venous blood was collected in heparinized sterile tubes. PBMC were separated by density gradient centrifugation on Ficoll-Hypaque (Histopaque, Sigma Aldrich, USA) after diluting with 1∶1 in RPMI 1640 (Sigma Aldrich) as described earlier [35]. Mononuclear cells were isolated by centrifugation at 800×g for 20 minutes; cells were washed three times in sterile 1× HBSS (GIBCO NY, USA) and re-suspended in RPMI 1640. Cell viability as estimated by 0.2% trypan blue (Sigma Aldrich) ranged from 95–98%.

### *Ex vivo* PBMC cultures

**Figure 1:**
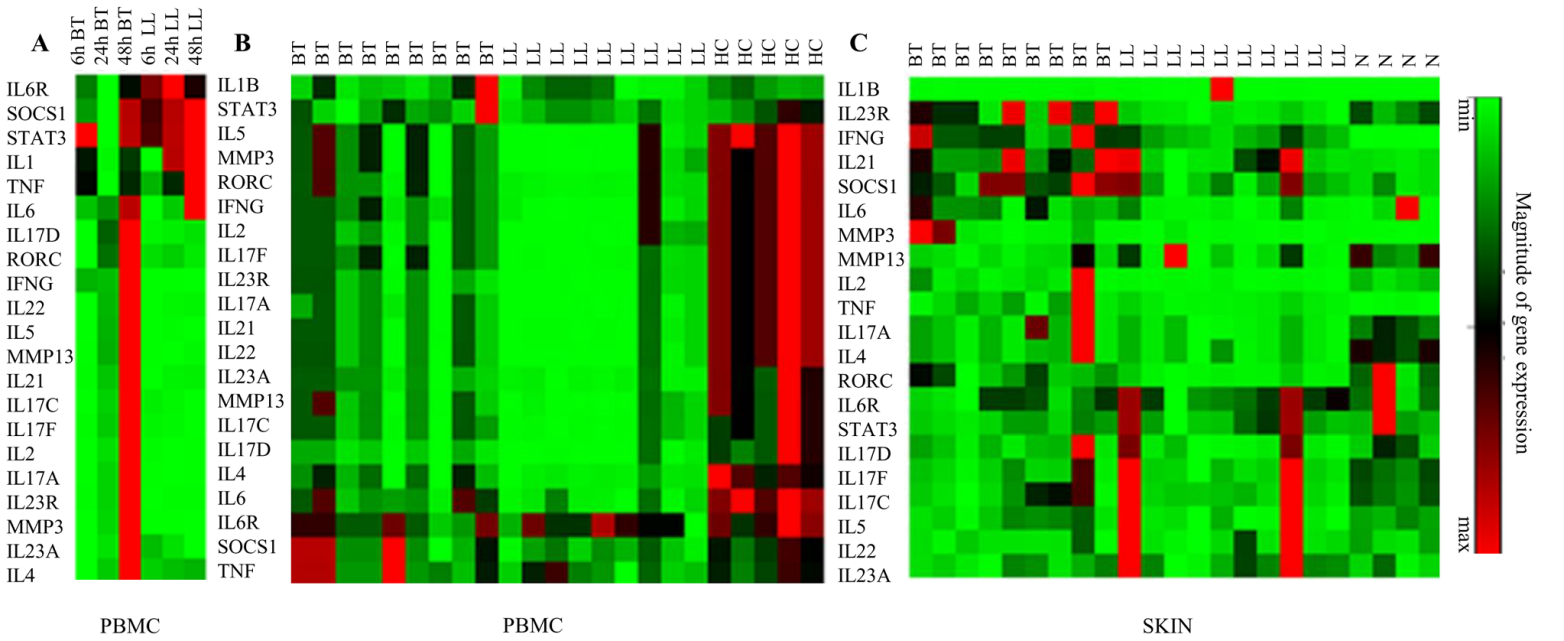
cDNA microarray heat map showing magnitude of expression. Fluoresce intensity of gene products determined by real time quantitative PCR (qPCR) with SYBER green as reporter. Selected cytokines, chemokines and transcription factors related to Th17 pathway and Th1 and Th2 cytokines are listed on the left side. Each horizontal row represents the same gene product and each vertical row each patient. The fluorescence range from high (red) to low (green) is indicated by the colored bar and reflects the degree of fluorescence intensity/gene expression. A. Shows time related gene expression at 6, 24 and 48 hrs of antigen stimulated PBMC in 3 each of borderline tuberculoid (BT) and polar lepromatous leprosy (LL) as described in Materials and Methods. B. Gene expression in 48 hr stimulated PBMC of 9 BT and 9 LL patients and 5 healthy contacts/controls (HC) exposed to long term infection showing the increased expression in HC followed by BT as compared to LL for the Th17 related genes. C. Skin lesions in general showed variability in some genes also show similar increased gene expression in HC, BT as compared to LL.

PBMC cultures were undertaken as described previously [35]. In brief, 1.5×10^6^ cells/ml suspended in RPMI 1640 (GIBCO NY,USA) with 10% pooled human AB serum, 2 mM L-glutamine, 100 units of penicillin ( Alembic Chemicals, India) and 100 ug streptomycin (Sarabhai Chemicals, India) were cultured in sterile flat bottom 24- well plates (Falcon, USA) as follows : Cells i) alone ii) with 5 ug/ml phytohemagglutinin (Sigma Aldrich) iii) with 10 ug/ml of *M leprae* sonicated antigen (ML) kindly provided by P J Brennan of Colorado State University. Initial studies on 3 each of BT and LL subjects were undertaken where cultures were incubated for 24, 48, and 72 h at 37°C in humidified 5% CO_2_+air. Subsequently all studies were undertaken at 48 h culture period at which time optimum results were obtained (Figure 1A).

After harvest, cells were washed as above and stored in RNA later (Sigma Aldrich) for gene expression studies or processed for flow cytometry analysis as given below. The paired supernatants were collected and stored at −80°C for ELISA.

### Skin biopsies

Sterile 4 mm punch (Cardiograph Co, Satara, Maharashtra, India) biopsies from skin lesions were obtained after application of 1% lignocaine (Kremoint Pharma, Maharashtra, India) as local anaesthesia. Part of the biopsy was processed in buffered formalin for routine histopathology. The reminder was placed in 1 ml of RNA later and stored at −80°C. Immediately prior to RNA isolation the frozen stored skin biopsies were thawed and the tissue crushed with liquid nitrogen in pestle and mortar.

### RNA isolation

RNA was isolated from both crushed skin tissue and PBMC cultures using RNeasy Mini Kit (Qiagen, Maryland, USA) according to the manufacturer's instructions. The isolated RNA was quantified using Nanodrop spectrophotometer (Nanodrop Technologies, Wilmington, USA) and purity at 260/280 from 1.8 to 2.0 was considered to be optimum. The quality of RNA was also checked for 28 s and 18 s RNA by electropherogram using Bio analyzer (Agilent Technologies, Inc, Singapore). Only samples with optimum RNA Integration Number value of ≥7 were used.

### Reverse Transcriptase PCR reaction

For cDNA synthesis 1 µg of total RNA was transcribed with RT First strand kit (SA Biosciences, MD, USA). Reactions were performed according to the manufacturer's instructions and the cDNA stored at −20°C till further use.

### Th17 PCR array

Gene expression was measured in quantitative real-time polymerase chain reaction (qPCR) using customized Th17 PCR array (SA Biosciences, Quiagen Co. CA, USA) as per the manufacturer's instructions. Duplicate samples of cDNA from each subject was amplified in 96 well plates containing primers for the genes of interest, cytokines and cytokine receptors: IFN-γ, IL-4, IL-5, IL-6, IL-6R, IL-2, IL-27, IL-17A,IL-17C,IL-17D,IL-17F, IL-1β, IL-21,IL-22, IL-23A, IL-23R; chemokines: MMP13,MMP3,CCL 20, CCL22; signaling molecules and transcription factors: RORC, SOCS1, STAT3; 5 housekeeping genes β2M, HPRT1, RPL13A, GAPDH, ACTB, using qPCR. 1 µg of cDNA was used per reaction in each well containing the ready to use PCR master mix and appropriate primers. These were then subjected to qPCR (ABI 7000, Applied Biosystems Singapore) for 2 h. Threshold cycles values were normalized and expressed as ΔCt: mean Ct of gene of interest-mean Ct of 5 housekeeping genes.

### Cellular and intracellular staining for flowcytometry analysis

All reagents were obtained from BD Biosciences, San Diego, CA. and used as per manufacture^r^'s instructions. For intracellular staining, *ex vivo* cultured cells were incubated with monensin (BD GolgiStop) for 8 h prior to harvest to block secretion of cytokine. For surface staining, 0.5×10^6^cells/50 ul in staining buffer were incubated with cocktail containing anti human CD3 (Per cpcy-5.5), CD4 (APC-H7) and CD8 (PE-Cy7) along with isotype controls of PE (mouse IgG1), Alexa Fluor 488 (mouse IgG1), Alexa Flour 647 (mouseIgG1) for 45 min at 4°C after which cells were washed two times and permeabilized with permeabilizing/fixation solution (containing saponin/paraformaldehyde) for 30 m at 4°C. The cells were washed two times and resuspended in Perm/Wash buffer and incubated with anti human IL-17A (Alexa Fluor-647), IL-17F (Alexa Fluor-488), and IL-21 (PE),) at 4°C for 30 min in the dark followed by two washes as before, resuspended in 500 ul. For evaluating phosphorylation of STAT3, cultured cells were first fixed for 10 min at room temperature, permeabilized as before with appropriate buffer and stained with a cocktail of PE labeled anti mouse STAT3, anti human IL-17A, CD3, CD4 and CD8 antibodies. Stained cells were acquired using BD FACS aria flow cytometry and analyzed with BD FACS Diva software.

### Estimation of cytokines by ELISA

Cytokines were estimated by ELISA (Ready Set Go, e-Bioscience, San Diego, CA, USA) in duplicate culture supernatants as per manufacturer's instructions. In brief, 96-well plates (Nunc, Rochester, NY, USA) were coated overnight at 4°C with capture biotin conjugated anti human antibodies for each of the cytokines, IL-17A/F, IL-21, IL-22, IL-23A, IL-6, IL-1β, IFN-γ and IL-5. Plates were washed 5 times, blotted and blocked with assay diluents for 1 h at room temperature. 100 µl/well of culture supernatant was added and plates incubated overnight at 4°C. After washing each well with buffer, appropriate avidin-horseradish peroxidase-conjugated anti-mouse antibody was added and the plates incubated at room temperature for 30 min. After washing as before, color development was undertaken using peroxidase color substrate TMB (Tetramethylbanzedine) and the reaction stopped by the addition of 1N H_2_SO_4_. The optical density (OD) of each well was read at 450 nm.

### Statistical analysis

Nonparametric statistics was performed using Graph Pad Prism version 5 (GraphPad Software, Inc., San Diego, CA, USA). Data were analyzed using two tailed Mann-Whitney for significance and Spearman tests for correlation coefficient. p≤0.05 was considered as statistically significant.

## Results

Both PBMC and skin biopsies were investigated in BT, LL patients and healthy subjects (Table 1) for expression of Th 17 related genes, phenotypic characterization of cells and cytokine release in *ex vivo* antigen stimulated PBMC cultures at 48 hrs which was found to be optimum in qPCR studies (Figure 1A).

### Th17 pathway genes show differential expression in clinical types of leprosy and healthy contacts

#### qPCR

**Figure 2:**
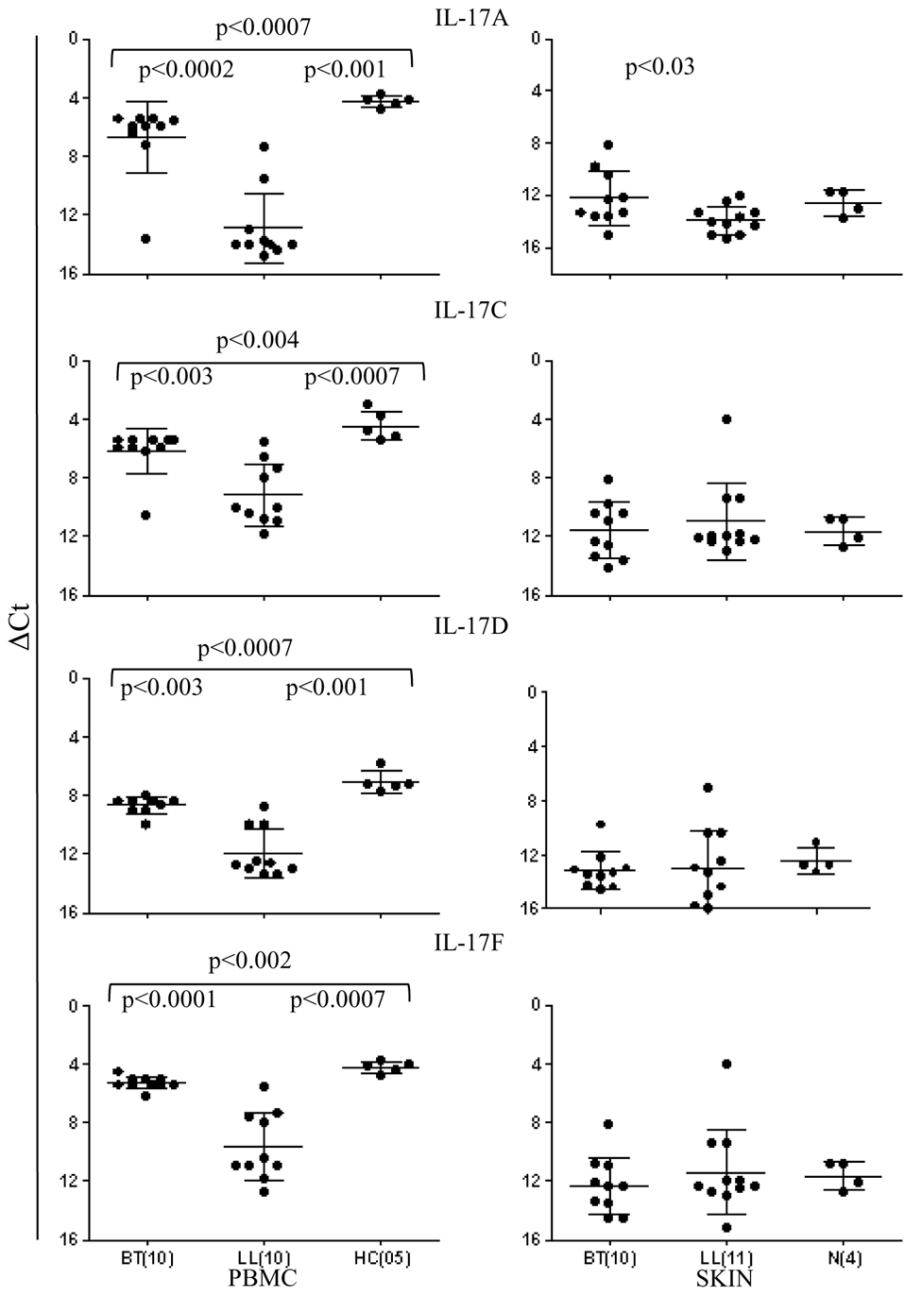
Scattergram of IL-17A, IL-17C, IL-17D and IL-17F expression (Mean ΔCt ± SD) in both antigen stimulated PBMC and skin lesions of BT, LL and HC subjects. Whereas, stimulated PBMC showed significant increase in all isoforms in BT and HC as compared to LL, the skin lesions showed lower expression with only IL-17A showing significant differences between BT and normal skin (N). p values given in the figure. Expression of IL-17A was marginally lower in LL without statistical significance. Numbers of subjects in each group is given in parenthesis. Abbreviations as in legend to Figure 1.

There was varying level of expression of IL-17A, IL-17C, IL-17D and IL-17F isoforms in ML stimulated *ex vivo* 48 h PBMC cultures in the three clinical groups with lowest levels for IL-17D (Figures 1B and 2). Individual variation was greater in LL for all the IL-17 isoforms. In general, the healthy contacts showed a significant increase in the expression of IL-17 isoforms as compared to the patient groups (p<0.0.002–0.0007) indicating that exposure to long term infection stimulated IL-17. Detection of clinical disease was associated with lowered IL-17 expression (Figure 2). It was not possible to study patients prior to onset of disease as leprosy has a long incubation period of years.

That the IL-17 release was also influenced by the leprosy spectrum was indicated by BT patients who showed significant increase in expression of IL-17 isoforms: IL-17A (p<0.0002), IL-17C (p<0.003), IL-17D (p<0.003) and IL-17F (p<0.0001) as compared to the multibacillary LL patients.

The status of IL-17 isoforms in skin biopsies (Figures 1C and 2) showed , lower expression of IL-17 isoforms in the skin of the three clinical groups as compared to antigen stimulated PBMC cultures ( p<0.001 to <0.0001). Furthermore, only IL-17A showed a significant increase in BT (Mean ΔCt ±SD, 12.2±2.0) as compared to the LL (Mean ΔCt ±SD, 13.91±1.05; p<0.03). Of interest was the expression of IL-17 in healthy skin of four subjects undergoing cosmetic surgery.

**Figure 3:**
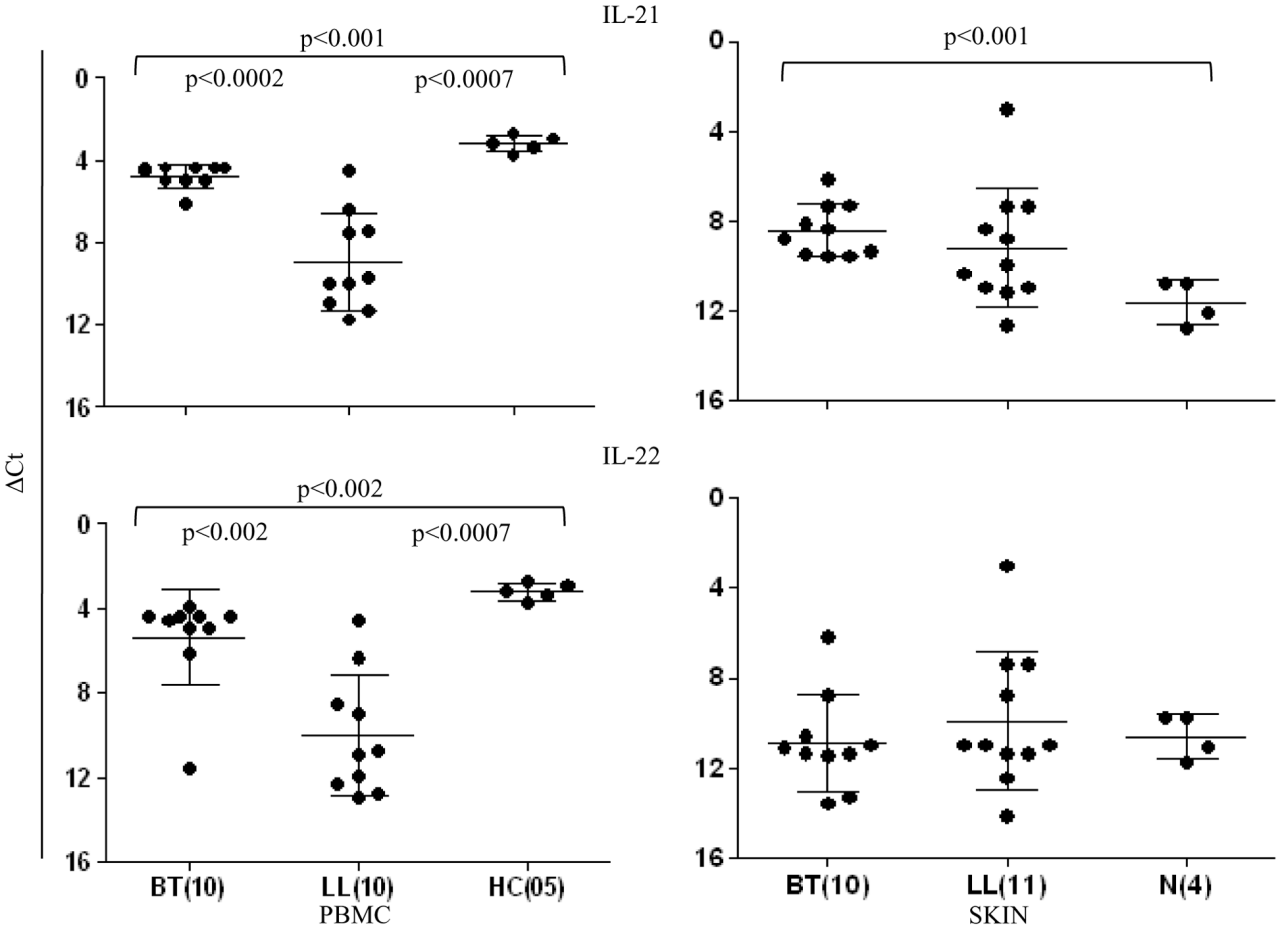
Scattergram of gene expression of IL-21, IL-22 (Mean ΔCt+SD) in PBMC and skin lesions of leprosy patients. Antigen stimulated PBMC showed significantly higher expression in BT as compared to LL for both cytokines (p values given in the figure). The skin lesions showed increase in IL-21 in leprosy patients as compared to normal skin. Abbreviations as in legends to Figures 1 and 2.

Cytokines IL-21 and 22 are known to be associated with IL-17 producing cells [25], [26]. Consistent with the above findings (Figures 1 and 3), both cytokines showed similar pattern (Figure 3). HC showed higher expression as compared to BT (p<0.002 ) and LL (p<0.0007)). Moreover, within the leprosy groups IL-21(p<0.002) and IL-22 (p<0.002) were significantly higher in BT as compared to LL type. Skin biopsies did not reveal differences in the leprosy types and only IL-21 showed significantly higher expression in dermal lesions of BT leprosy as compared to normal skin samples (p<0.001).

#### IL-17 is produced by CD4^+^ T cells

**Figure 4:**
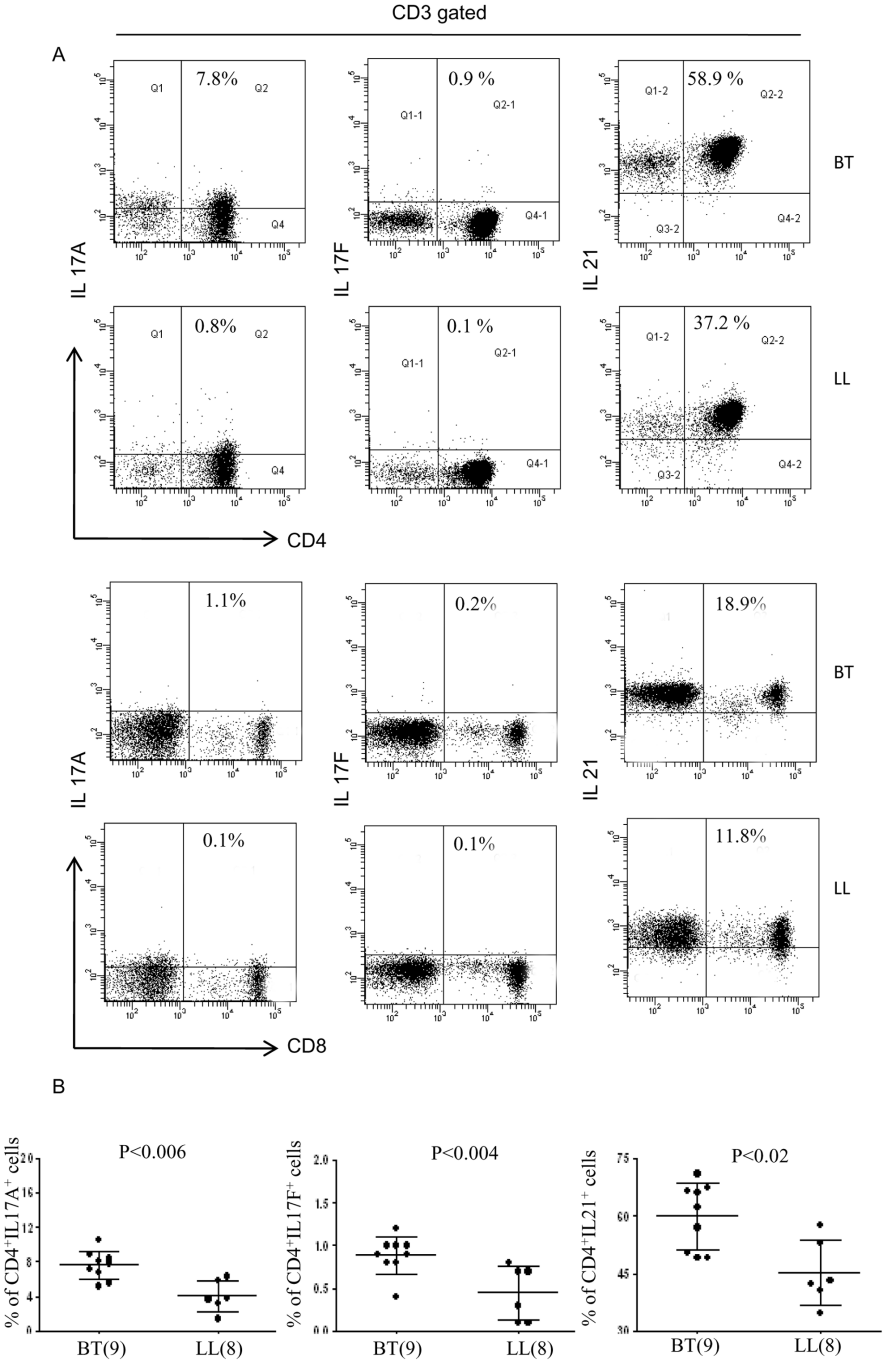
IL-17A, IL-17F and IL-21 producing cells are predominantly of CD4^+^ T cell lineage in leprosy. A. Representative data from one each of BT and LL patients of antigen stimulated PBMC using multi-color flowcytometry (see Material and Methods). The CD3 gated population showed intracellular cytokines predominantly in the CD4^+^ cells. Percentage of IL17A^+^ cells was higher than IL17F^+^ cells. % of IL21^+^ cells was highest than the others in both types of leprosy. CD8^+^ cells also showed IL-21 in a smaller percentage of cells as indicated in the figure. BT showed higher percentage of all there cytokines as compared to LL subjects. % of cells is given in the upper quadrant of the figure B. Scattergram depicting Mean % ± SD of the cytokine bearing cells in 9 BT and 6 LL subjects, confirming the significant increase in percentage of cells in BT for all 3 cytokines as indicated by p values given in the figure. Abbreviations as in Figure 1. Parenthesis shows number of subjects tested and numbers in figure give % of double positive cells.

Flowcytometry analysis of PBMC cultures showed IL-17A and F to be associated with CD3^+^CD4^+^ cells (Figure 4A). Consistent with above findings BT patients had significantly higher (p<0.006) numbers of CD4^+^ IL-17A^+^ cells (Mean %± SD 7.8±1.6) as compared to anergic LL (Mean ±SD: 4.0±1.9). Though IL-17F^+^ cells were <1% of the CD4^+^ cells nevertheless they showed statistically significant decrease in LL patients (p<0.004). CD8^+^ cells with IL-17^+^ were ≤0.2% in the leprosy patients. It is of interest that the same subjects also showed higher percentage of IL-21^+^ cells in both types of leprosy. Moreover, they were significantly higher in BT (p<0.02) as compared to the LL group. Unlike IL-17, IL-21 was present in both CD4^+^ and CD8^+^ T cells in the two clinical types of leprosy.

#### IL-17A+ cells show association with CCR6

**Figure 5:**
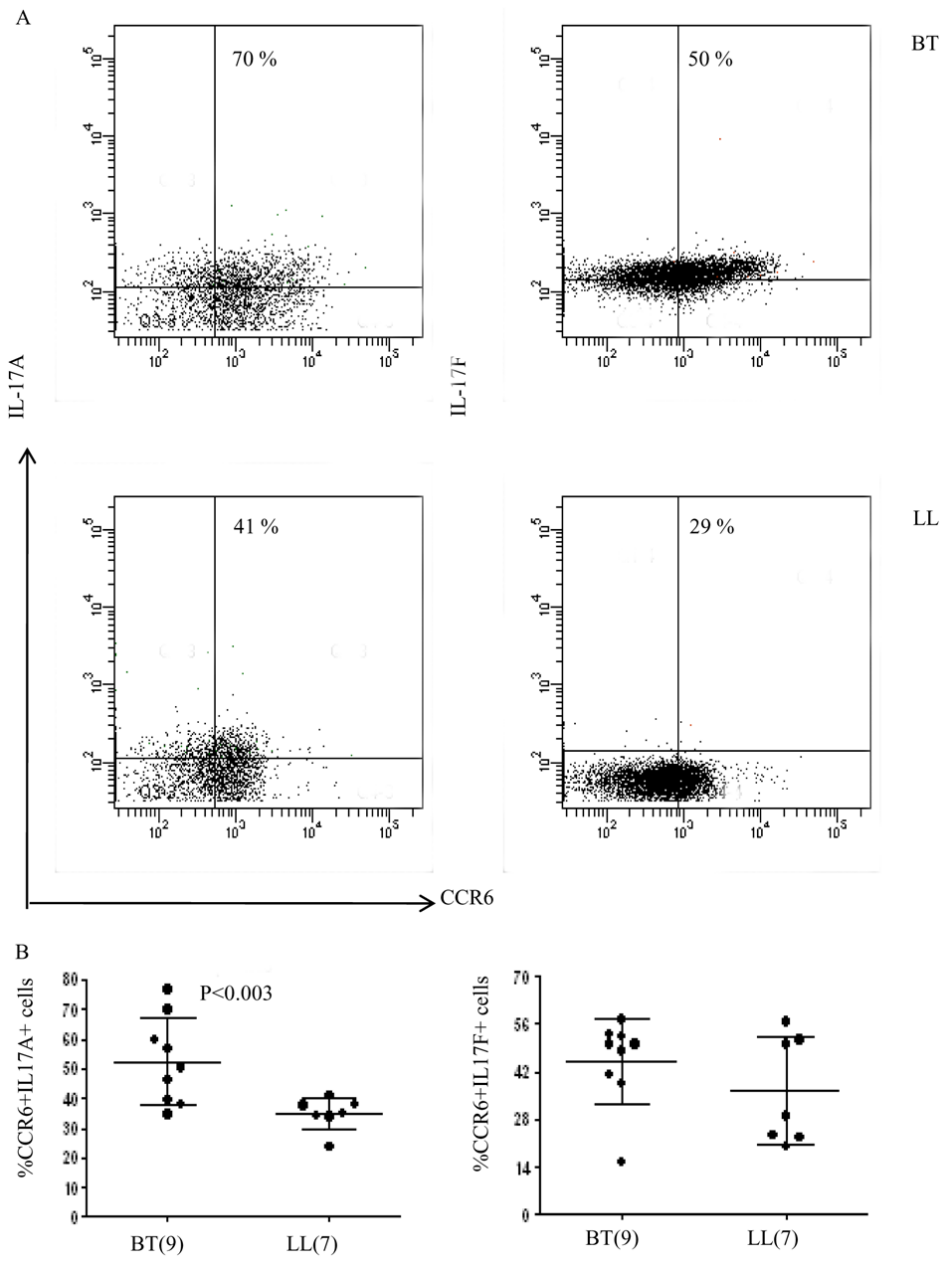
IL 17 producing cells belong to CD3^+^CD4^+^CCR6^+^ effector T cell lineage. A. Representative flow cytometry analysis on antigen stimulated PBMC of one each of BT and LL patients (see Materials and Methods). CD3^+^ gated CD4^+^ cells were examined for the presence of intracellular IL-17 and surface CCR6 (see Materials and Methods). As may be seen both IL-17A and IL-17F containing cells showed CCR6. The % of IL 17A/F^+^ CCR6^+^ cells was higher in BT as compared to LL as indicated in the figure. % of cells is given in the upper quadrant of the figure. B. Scattergram of BT and LL subjects showing Mean%+SD of IL-17A/F^+^CCR6^+^ cells which was significantly higher for IL-17A in BT as compared to LL as indicated by p values given in the figure. Abbreviations and legend as in figure 4.

We next investigated the association of CD4^+^ IL17^+^ cells with CCR6, a marker of effector T cells [36]. It may be seen from Figures 5A and 5B that CCR6 was associated with both IL-17A^+^ and IL-17F^+^ cells. In general, the mean percentage of these cells were as expected higher in BT as compared to LL subjects with only 1L-17A^+^ cells showing statistical significance (p<0.003). Thus it would appear that a large proportion of IL-17A^+^ cells have effector T cell lineage.

#### ELISA

That IL-17 mRNA transcribed to the IL-17 protein was confirmed in supernatants of ML stimulated PBMC which showed significantly higher IL-17A/F in BT (p<0.0005) as compared to LL patients (Table 2). IL-21 but not IL-22 also showed significant increase in BT as compared to LL (p<0.003).

**Table 2:** Mean pg/ml ± SD and range of cytokines in culture supernatants of 48 hr MLSA stimulated PBMC from TT/BT and LL patients.

Cytokines	Detectable level (pg/ml)	pg/ml			
		BT (10)		LL (11)	
		Mean ± SD	Range	Mean ± SD	Range
IL-17A/F	30	101.9±26.28***	45–169.7	45.5±22.07	0–111
IL-21	30	314.2±19.18***	280–341	267.4±11.5	245–286
IL-22	8	561.4±118	399–782	633.1±89.18	489–748
IL-23	15	62.65±51.25**	23–225	22.5±11.4	0–52.6
IL-6	2	50.5±27.9***	2.5–94	21.8±10.9	2–42.7
IL-1β	4	13.6±16.34	4–66	8.58±4.4	4–19.8
IFN-γ	4	749.7±1314**	58.4–4870	53.12±23.3	6–90
IL-5	4	9.24±9.39	0–26	36.4±14.46***	23–62.7

BT; borderline tuberculoid leprosy respectively, LL; polar lepromatous leprosy, as per Ridley Jopling classification [2] MLSA: heat killed sonicated armadillo derived *M.leprae* antigens.

** p<0.001,

*** p<0.0001 by two tailed Mann Whitney test. 0: below detectable level.

Taken collectively the above data shows that CD4^+^IL-17^+^ CCR6^+^ cells are present in circulation of leprosy subjects. At the site of the dermal lesions IL-17 was expressed at lower levels as compared to PBMC cultures and IL-17A was the more discriminatory isoform in leprosy lesions.

### Transcription factors support Th17 signature in leprosy

**Figure 6:**
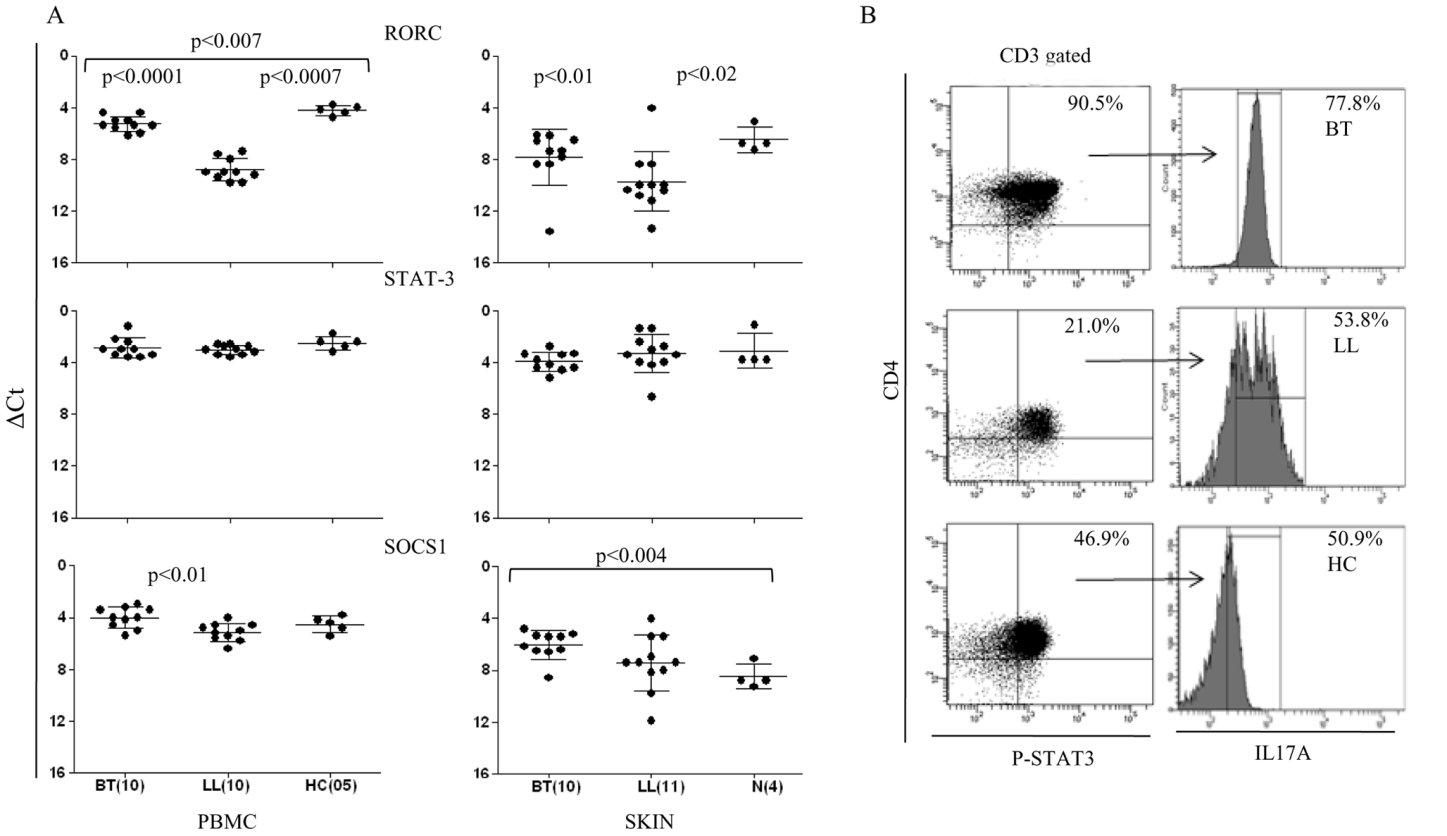
RORC, STAT3, SOCS1 expression in antigen stimulated PBMC and Skin lesions of leprosy and healthy subjects. A. Scattergram of Mean ΔCt ± SD of gene expression shows significant increase in RORC in healthy as compared to BT, Within leprosy types BT showed significantly higher RORC expression as compared to LL. Expression of STAT3 was similar in the 3 groups, and SOCS1 showed moderate and significance increase in BT as compared to healthy contacts as indicated by the p values in the figure. B. Phosphorylation of STAT3 is associated with intracellular IL-17 A in CD4^+^ cells as shown by multi-color flow cytometry analysis of antigen stimulated PBMC. Of the P-STAT3 cells BT showed higher % of IL-17A^+^ cells as compared to LL and healthy subjects as indicated in the upper quadrant. Abbreviations and legend as in figure 4.

The role of transcription factors RORC and STAT3 associated with Th17 cells was explored. Figure 6A shows not only association of Th17 cells with RORC but also its increased expression in both HC and BT as compared to LL patients in the antigen stimulated PBMC cultures (p<0.0007 and p<0.0001 respectively) and the dermal lesions (p<0.02, p<0.01 respectively). It also showed strong correlation with IL-17A and IFN-γ in PBMC cultures (r^2^ = 0.94 and 0.84 respectively, p<0.0001) of both types of leprosy.

STAT3 on the other hand showed high expression but did not discriminate between the clinical groups (Figure 6A) nor showed correlation with IL-17 expression. With a view to further dissect the role of STAT3 we next studied its phosphorylation status in 3 subjects each of BT, LL and HC. Figure 6B presents representative data on CD3^+^ gated cells, wherein CD4^+^ cells of antigen stimulated PBMC showed phosphorylated STAT3. The highest percentage of cells was in BT (85.1 to 90.58%) followed in decreasing order by HC (45.8 to 46.9%) and LL (19.2 to 21.0%) subjects. More importantly, in all clinical groups STAT3 phosphorylation was associated with >50% of CD4+IL-17^+^ cells (Figure 6B) with BT showing the highest percentage (69.2 to77.8%). One of the known promoters of phosphorylation and activation of STAT3 signaling pathway is SOCS1 [37], [38] which also showed higher expression in BT (p<0.01) as compared to LL patients in PBMC cultures. In skin lesions it was higher in BT when compared to normal skin (p<0.004) (Figure 6A). In summary, it is evident that RORC was associated with IL-17+ cells and phosphorylated STAT3 rather than total STAT3 expression revealed functional differences in leprosy and was in agreement with the activation by SOCS1.

### Role of cytokines involved in development and differentiation of Th17 cells in leprosy

Cytokines IL-1β, IL-23 and IL-6 reported to influence and sustain Th17 lineage [39], [40] were investigated. In general, the expression of all cytokines was higher in *ex vivo* antigen stimulated PBMC as compared to skin lesions reflecting differences due to recall responses in antigen stimulated PBMC as compared to the status of an ongoing *in vivo* response.

IL-1β, showed high expression in all subjects without discriminating the leprosy types in PBMC cultures. Mean ΔCt± SD ranged from 2.6±1.4, 3.1±1.3, 2.3±0.37 respectively in BT, LL and HC. It is of interest that BT skin lesions showed statistically significant increase in expression of IL-1β (p<0.008) as compared to normal skin.

**Figure 7:**
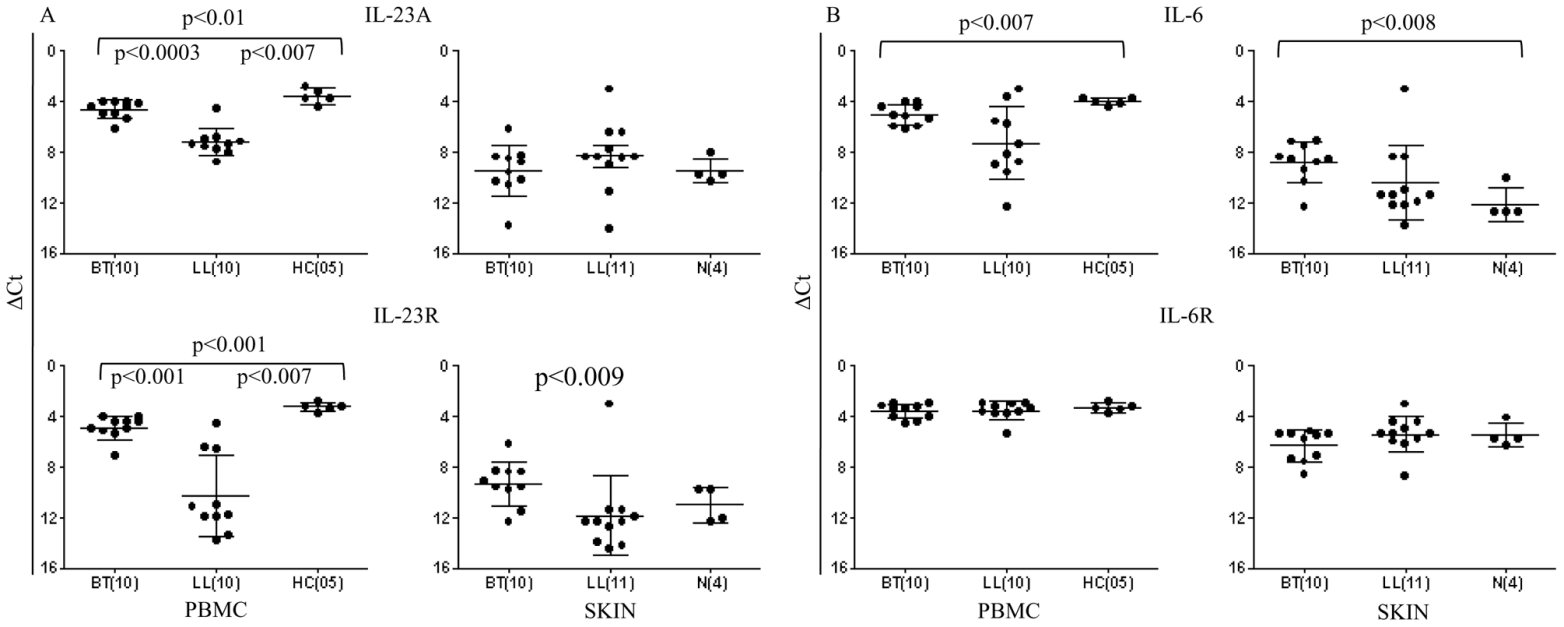
Scattergram showing expression of cytokines IL-23, IL-6 and their respective receptors. Mean ΔCt ± SD of gene expression in antigen stimulated PBMC and skin from leprosy and healthy subjects. IL-23 and IL-23R showed significantly higher expression in BT and healthy as compared to LL subjects. IL-6 showed higher expression in healthy as compared to BT as indicated in the figure. In the normal (N) skin, IL-6 expression was significantly lower as compared to BT. Abbreviations and legend as in figure 2.

In contrast, IL-23A considered to be important for maintenance of Th17 cells [41] showed significant increase in HC and BT (p<0.01) groups as compared to LL (p<0.007, Figure 7, Table 2). Moreover, IL-23A was significantly higher in BT (p<0.0003, Mean ΔCT ±SD ΔCt: 4.7±0.7) as compared to LL (Mean ΔCt ±SD: 7.26±1.09) indicating differential expression within the two leprosy types. ELISA confirmed, IL-23 protein to be significantly higher in the antigen stimulated PBMC supernatants of BT as compared to LL patients (p<0.001, Table 2). Surprisingly, the findings in the skin for IL-23A expression did not show discrimination between the clinical leprosy types.

In agreement with the above, IL-23R (Figure 7) which is considered to be important for stabilization of Th17 cells also showed increase in both antigen stimulated PBMC and skin of BT group as compared to LL (p<0.001 and p<0.009 respectively). Moreover, IL-23R showed significant correlation with IL-17A (r^2^ = 0.58 p<0.008), IL-17F (r^2^ = 0.52 p<0.02), IL-21 (r^2^ = 0.64 p<0.003), IL-22 (r^2^ = 0.58 p<0.008) and IFN-γ (r^2^ = 0.59 p<0.007).

IL-6 expression also showed a dichotomy between dermal lesions and PBMC cultures of BT patients. Whereas it showed decrease in patients as compared to HC (p<0.007), dermal lesions showed increase as compared to the healthy skin (p<0.008). No distinction was seen between the leprosy types. However, IL-6 ELISA showed significantly lower levels in LL as compared to BT (p<0.0001) in culture supernatants. Taken together our data supports the greater role of IL-23 and its ligand IL-23R in leprosy as compared to IL-6/IL-6R for IL-17 production in both circulating cells and at the site of the dermal lesions.

IL-27 and IL-2 have been reported to negatively regulate Th17 cells [42], [43]. Paradoxically, the expression of these cytokines was also higher in healthy contacts as compared to leprosy types (p<0.001) but did not show differences within the leprosy groups even though expression was lower in LL (Table 3). These cytokines did not show significant correlation with IL-17 isoforms (Spearman tests) indicating that other regulatory factors may be involved in leprosy.

**Table 3:** Mean Δ ct ± SD of cytokines (negative regulator of Th17 cells differentiation) of 48 hr MLSA stimulated PBMC and skin from BT, LL patients and healthy contacts.

	PBMC			SKIN		
	BT	LL	HC	BT	LL	HC
IL-27	6.86±1.5	7.94±2.3	5.16±0.47** a	11.94±2.75	8.6±2.6* b	9.2±1.4
IL-2	6.97±2.5	8.54±4.2	4.28±0.36** a	10.5±1.9** b	12.87±3.7	10.53±1.09* c

BT: borderline tuberculoid leprosy, LL: polar lepromatous leprosy, as per Ridley Jopling classification [2], HC; healthy contacts exposed to leprosy patients to 1–2 years.

* ; p<0.05,

** ; p<0.001(Mann Whitney test),

a ; BT vs HC,

b ; BT vs LL,

c ; LL vs HC.

### Th17 associated chemokines show differential expression in skin lesions

**Figure 8:**
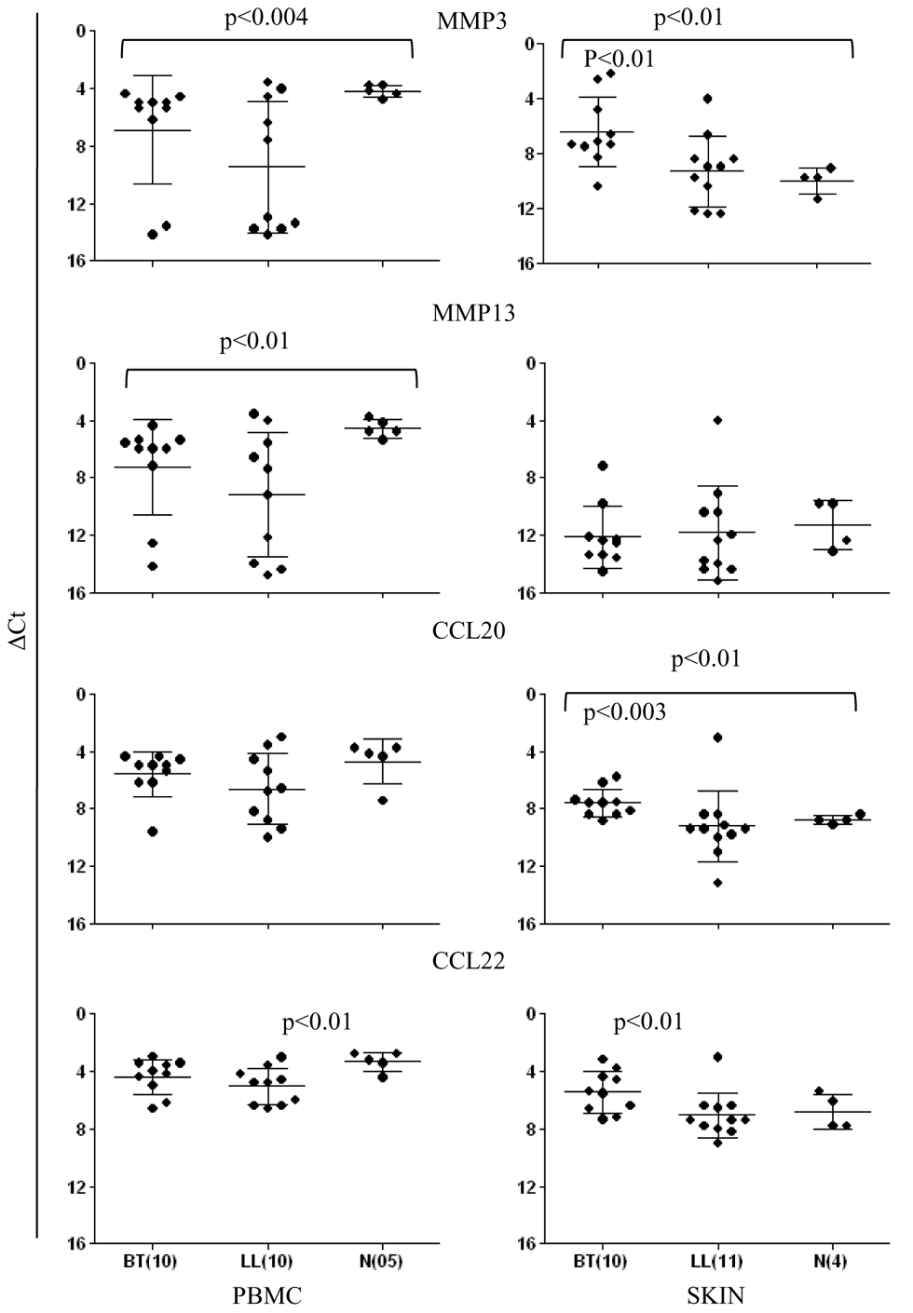
Scattergram showing expression of chemokines MMP3, MMP13, CCL20 and CCL22 in leprosy and healthy subjects in antigen stimulated PBMC and skin. Whereas PBMC did not show differences within the leprosy groups for the chemokines (Mean ΔCt ±SD) at the site of the skin lesions MMP3 showed significantly higher expression in BT. Differential expression for the chemokines was observed between healthy skin and BT lesions for some of the chemokines ( p values given in the figures). Abbreviations and legend as in figure 2.

As shown in Figure 8, expression of MMP3, MMP13, CCL22 were seen to be highest in PBMC cultures of healthy contacts as compared to leprosy types. The former two chemokines showed statistically significant decrease in BT (p<0.004, p<0.01 respectively). CCL22 showed decrease in LL as compared to healthy subjects (p<0.01) but did not discriminate the leprosy types. In contrast, skin lesions showed distinct differences in chemokine expression. MMP3, CCL20 and CCL22 showed increase in BT as compared to LL lesions (p<0.01, p<0.003, p<0.01 respectively). Of interest, was the higher expression of MMP3 and CCL20 in BT as compared to normal skin suggestive of a possible role in the trafficking of relevant cells to the sites of tuberculoid leprosy granulomas.

### IL-17 expression is related to the state of Th polarization in leprosy

**Figure 9:**
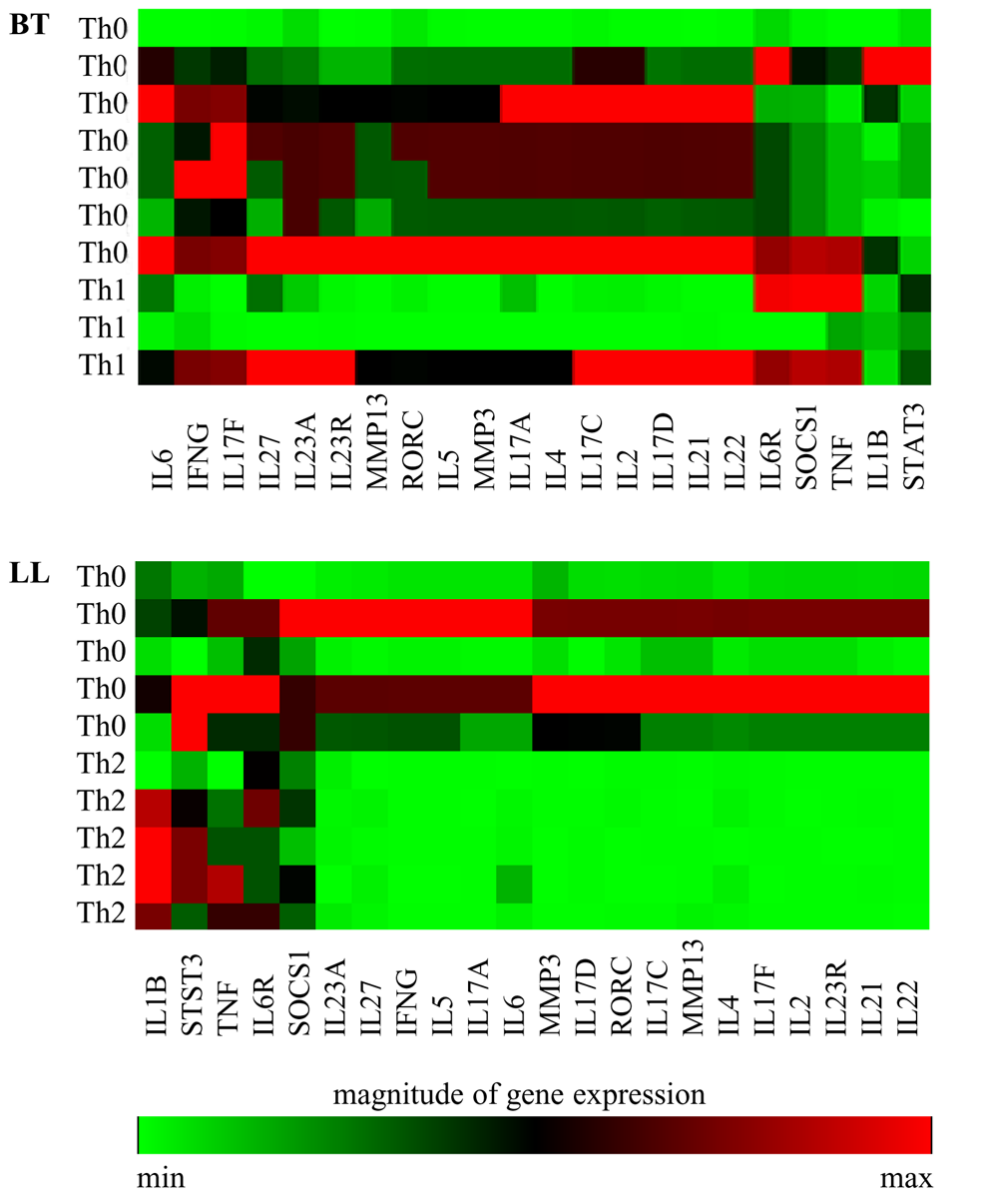
cDNA microarray heat map showing magnitude of expression. Range of expression is indicated by fluoresce intensity of gene products following real time PCR (q PCR), for selected cytokines, chemokines and transcription factors related to Th17 pathway and Th1 and Th2 cytokines as given in Figure 1 and listed at the bottom. Each vertical row represents the same gene product and each horizontal row each patient whose Th status is indicated on the right of the image. Magnitude of gene expression of Th17 related genes is depicted as intensity of fluorescence in 10 each of BT and LL patients who have been classified on the basis of polarized Th1, Th2 and non discriminating Th0 cytokine profile. Patients with the Th0 cytokine profile showed higher expression of the IL-17 related genes as compared to Th1 and Th2 defined patients. Legend and bar as in Figure 1.

We next investigated the relationship of Th17 cells to the Th polarization status in leprosy. The status of Th subsets in individual subjects is provided by Figure 9 showing the fluorescent signal as an indicator of the magnitude of gene expression. Tables 4 and 5 give ΔCt values of IFN-γ and IL-4 to assign Th status. Expression of Th17 related genes in the same subjects was graded arbitrarily from nil (−) to (+++) to illustrate more easily their relationship with Th status. It may be observed that IL-17A, IL-17F, IL-21 and IL-22 were expressed more in the non-polarized Th0 group of patients in both types of leprosy (Table 4) than in subjects with Th1 (p<0.01) and Th2 (p<0.01) subsets (Table 5). Seven of the 10 BT patients showed Th0 as indicated by the expression of both IFN-γ and IL-4. The same patients also showed +++ expression of IL-17A, IL-17F, IL-21, IL-22 and IL-23R. Transcription factor RORC showed moderate ++ expression in Th0. In contrast, Th1 polarized patients showed low/nil expression (Figure 9 and Table 5). In lepromatous leprosy 5 each of 10 patients were of Th2 and Th0. As may be noted the latter group again showed significantly higher expression levels of Th17 related genes as compared to the polarized Th2 (p<0.01 ). However, it may be pointed out that expression of the Th17 related genes in LL was lower (++) as compared to BT (+++) in the Th0 groups (Table 4). RORC again showed lower level of expression as compared to the Th17 cytokines but was similar expression (+) in Th0 and Th2 subsets in LL patients. It is of interest that IL-17 was also associated with IFN-γ in tuberculoid leprosy patients who had Th0 cytokine profile suggesting that both cytokines are involved in the T cell immune responses. Consistent with the above findings in BT, 5 HC who had Th0 profile also showed enhanced expression of Th17 related genes.

**Table 4:** IL-17 and relevant cytokines, IL-21, IL-22 , receptor IL-23R and transcription factor RORC in leprosy patients with Th phenotypes.

	BT	BT	BT	BT	BT	BT	BT	LL	LL	LL	LL	LL	HC	HC	HC	HC	HC
IL4 (ΔCt)	5.4	4.4	5	4.6	4.4	6.2	5	5.6	3.6	7.6	4.1	7.4	3.2	3.8	4.4	3.8	8.4
IFNγ(ΔCt)	5.4	5.4	5	4.6	5.4	6.2	6	4.6	3.6	8.6	3.1	7.4	4.2	4.8	4.4	3.8	7.4
Th type	Th0	Th0	Th0	Th0	Th0	Th0	Th0	Th0	Th0	Th0	Th0	Th0	Th0	Th0	Th0	Th0	Th0
IL-17A	+++	+++	+++	+++	+++	+++	+++	++	++	++	++	++	+++	+++	+++	+++	++
IL-17C	+++	+++	+++	+++	+++	+++	+++	++	++	++	++	++	+++	++	++	++	++
IL-17D	++	++	++	++	++	+	++	++	++	++	++	++	+++	++	++	++	++
IL-17F	+++	+++	+++	+++	+++	+++	+++	++	++	++	++	++	+++	+++	+++	+++	++
IL-21	+++	+++	+++	+++	+++	+++	+++	++	++	++	++	++	+++	+++	+++	+++	++
IL-22	+++	+++	+++	+++	+++	+++	+++	++	++	++	++	++	+++	+++	+++	+++	++
IL-23R	+++	+++	+++	+++	+++	+++	+++	++	++	++	++	++	+++	+++	+++	+++	++
RORC	++	++	++	++	++	++	++	+	+	+	+	+	++	++	++	++	++

Subjects with non polarized Th 0 showed increased expression as compared to polarized Th 1 and Th 2 in all clinical groups as shown in Table 5.

Mean Ct of 5 housekeeping genes varied from 21–22.9; Th1 and Th2 were defined on the basis of expression of IFN-γ and IL-4 given as Δ Ct in the upper two rows. IL-17 related gene expression was arbitrarily graded to indicate the range of expression on the basis of Δ Ct of each gene product: (−): 15 to 20 (Ct above 35 cycles was considered negative). (+): 10 to 14, (++): 7 to 9, (+++) 4 to 8. BT: borderline tuberculoid; LL: polar lepromatous leprosy. HC; healthy contacts exposed to leprosy patients to 1–2 years.

**Table 5:** IL-17 and relevant cytokines, IL-21, IL-22, receptor IL-23R and transcription factor RORC in leprosy patients with Th1 and Th2 phenotypes showing decreased expression of Th17 related genes as compared to Th0 given in Table 4.

	BT	BT	BT	LL	LL	LL	LL	LL
IL4 (ΔCt)	13	14	8.8	8.2	7.8	8.8	8	8.4
IFNγ(ΔCt)	8.2	8.6	7.8	12	14	13	13	13
Th type	Th1	Th1	Th1	Th2	Th2	Th2	Th2	Th2
IL-17A	+	−	−	−	−	+	+	−
IL-17C	+	+	+	+	+	+	+	+
IL-17D	−	−	−	+	+	+	+	+
IL-17F	−	+	+	+	+	+	+	+
IL-21	+	+	+	+	+	+	+	+
IL-22	−	+	−	+	+	+	+	+
IL-23R	+	+	+	+	+	+	+	+
RORC	+	+	+	+	+	+	+	+

Legend as in Table 4.

In conclusion, it would appear that Th17 cells form a third Th subset of CD4^+^ effector cells in leprosy in addition to the Th1 and Th2 types.

## Discussion

Th17 cells have emerged as a third subset of Th cells that play an important role not only in autoimmune diseases where they were first described but also in many infectious diseases in man and in experimental models [14], [15], [16], [17]. The present investigation was undertaken on leprosy patients with a view to understanding the immune mechanisms underlying the unique clinical types in the leprosy spectrum as well as in investigating the differences between circulating cells and local immune responses in the lesional skin. In general, expression of IL-17 isoforms in skin lesions was lower than in antigen stimulated PBMC which may be related to the differences in numbers of cells in lesions or the stronger recall responses in circulating cells. Of interest was the finding that healthy household contacts with long term exposure to the patients showed the highest IL-17 expression as compared to the diseased subjects suggesting that IL-17 was an important early adaptive immune response to *M.leprae*. Within the leprosy spectrum the more resistant paucibacillary form of tuberculoid leprosy had higher IL-17 associated cytokines IL-21, IL-22 and the transcription factor RORC. The signatures of Th17 pathway genes were further endorsed by cytokine secretion into the supernatants of PBMC cultures (Table 2). Consistent with this was also the increased expression of IL-23 and IL-23R known to be involved in differentiation of Th17 cells. It appears that IL-23R was more relevant in leprosy than IL-6R reported to function in a similar manner [44], [45] indicating that the pathway of Th17 may show subtle differences not only in man as compared to the mouse but also in different diseases. STAT3 expression *per se* was uniformly strong and did not show differences between the 3 clinical groups in the PBMC or skin lesions. Importantly, phosphorylated STAT3 was much higher in tuberculoid as compared to the other two groups revealing functional and signaling differences in them. SOCS1 known to be a regulator of Th17 differentiation also showed significantly increased expression (p<0.01) in the resistant form of leprosy and supported the increased activation of STAT3 in tuberculoid leprosy. Chemokines associated with Th17 cells were found to be discriminatory more at the local level in dermal lesions of the two clinical types of leprosy than in PBMC cultures. MMP3, CCL20 and CCL22 showed significance increase in tuberculoid as compared to the lepromatous lesions adding support to the increased lymphocytes seen in BT granulomas. Due to lack of evidence by histochemistry we were unable to formally establish Th17 lineage of the lesional lymphocytes. Using flowcytometry, we determined that a large proportion of IL-17^+^ cells in antigen stimulated PBMC cultures were CCR6^+^ suggestive of CD4^+^ effector T cell population [36].

Our findings in leprosy are similar to reports in cutaneous leishmaniasis [16], tuberculosis [15] and inflammatory bowel disease [20] where Th17 cells were shown to regulate the immune responses in man. The emergence of Th17 cells during leprosy reactions of type 2 or erythema nodosum leprosum has been reported to play an important part in the inflammatory component seen in these states known to be associated with lepromatous leprosy [46]. A recent study on Brazilian populations indicated reduced expression of IL-17A in leprosy skin as compared to healthy subjects and attributed it to genetic differences [47]. The differences between our and their findings may be related to differences between the two ethnically diverse populations. That *Candida albicans* and *Staphylococcus aureus* induce Th17 cells that produce different cytokines such as IFN-γ or IL-10 in addition to IL-17 would suggest that regulation of these cells is not only pathogen dependant but also due to other factors such as cytokines and transcription factors [48]. In human tuberculosis, predominance of IL-22 over IL-17 has been seen at the site of disease [49], a feature that was not observed in the skin lesions of leprosy. In our study it is evident that IFN-γ was produced in the same PBMC cultures concomitantly with IL-17 in healthy contacts and tuberculoid leprosy. IL-23A played a more important role than IL-6 for Th17 differentiation in leprosy which is reminiscent of the report on *Mycobacterium bovis* infection in a murine model where IL-17 was observed from day 1 of infection, was dependant on IL-23 and associated with granuloma formation [14].

We also explored the relationship of Th17 cells to the conventional Th1 and Th2 subtypes of T cells. Earlier reports [8], [9], [50] including ours had shown polarized Th1 and Th2 phenotypes in PBMC cultures of tuberculoid and lepromatous leprosy respectively. This had become a paradigm in explaining the converse pattern of CMI to antibody responses observed in leprosy spectrum. However, the finding of non polarized Th0 phenotypes in many patients of both types of leprosy required further explanation [9]. Therefore, we analyzed the status of Th17 pathway against the Th1 and Th2 background. It is evident from Figure 9 and Tables 4 and 5 that Th17 cells were strongly associated with the non polarized Th0 phenotype in both leprosy types and healthy subjects who showed concomitant IFN-γ and IL-4/IL-5 both by qPCR and ELISA of PBMC culture supernatants. It would appear that Th17 cells form a third subset of Th cells and may play an important role in those patients where Th1 and Th2 polarization is not observed. Taken together the data indicates that in addition to IFN-γ producing Th1 cells, CD4^+^IL-17^+^ cells have a role in the adaptive immune response in the more resistant form of tuberculoid leprosy and discriminate between the clinical types of leprosy. However, the Th17 distinction between the paucibacillary BT and the multibacillary LL were less obvious in polarized Th1 and Th2 states. It is possible that Th17 lineage may be an alternate pathway for bacillary clearance when the patient is unable to mount Th1 response or when Th polarization has not set in. Supportive of this were the results obtained on subjects exposed to long term infection who continued to be healthy and not develop the disease. It is not possible from this one time point investigation to conclude whether Th17 indicates T cell plasticity or constitutes a stable lineage. It is of interest that, vaccination against *Mycobacterium tuberculosis* lung infection produced protection which was associated with IL-23A and IL-17 responses where IL-17 preceded IFN-γ responses [51]. Whether Th17 is a rescue pathway or is a stable alternate defense immune mechanism needs further investigation. In the absence of suitable animal models which mimic the leprosy spectrum, investigations during disease states can only provide limited information on the dynamics of an immune response. Nevertheless as far as we know this is the first report in leprosy that provides evidence for the presence of Th17 cells as a third Th subset of the adaptive immune mechanism in subjects who have not developed the conventional Th1 and 2 phenotypes.
